# Immobilization and Characterization of L-Asparaginase over Carbon Xerogels

**DOI:** 10.3390/biotech11020010

**Published:** 2022-04-14

**Authors:** Rita A. M. Barros, Raquel O. Cristóvão, Sónia A. C. Carabineiro, Márcia C. Neves, Mara G. Freire, Joaquim L. Faria, Valéria C. Santos-Ebinuma, Ana P. M. Tavares, Cláudia G. Silva

**Affiliations:** 1 LSRE-LCM—Laboratory of Separation and Reaction Engineering-Laboratory of Catalysis and Materials, Faculty of Engineering, University of Porto, 4200-465 Porto, Portugal; up201604653@edu.fe.up.pt (R.A.M.B.); roc@fe.up.pt (R.O.C.); sonia.carabineiro@fct.unl.pt (S.A.C.C.); jlfaria@fe.up.pt (J.L.F.); 2 ALiCE—Associate Laboratory in Chemical Engineering, Faculty of Engineering, University of Porto, 4200-465 Porto, Portugal; 3 LAQV-REQUIMTE, Department of Chemistry, NOVA School of Science and Technology, Universidade NOVA de Lisboa, 2829-516 Caparica, Portugal; 4 CICECO-Aveiro Institute of Materials, Department of Chemistry, University of Aveiro, 3810-193 Aveiro, Portugal; mcneves@ua.pt (M.C.N.); maragfreire@ua.pt (M.G.F.); 5 Department of Engineering Bioprocess and Biotechnology, School of Pharmaceutical Sciences, UNESP-University Estadual Paulista, Araraquara 14800-903, Brazil; valeria.ebinuma@unesp.br

**Keywords:** L-asparaginase, enzyme immobilization, carbon xerogels, physical adsorption, central composite design

## Abstract

L-asparaginase (ASNase) is an aminohydrolase currently used in the pharmaceutical and food industries. Enzyme immobilization is an exciting option for both applications, allowing for a more straightforward recovery and increased stability. High surface area and customizable porosity make carbon xerogels (CXs) promising materials for ASNase immobilization. This work describes the influence of contact time, pH, and ASNase concentration on the immobilization yield (*IY*) and relative recovered activity (*RRA*) using the Central Composite Design methodology. The most promising results were obtained using CX with an average pore size of 4 nm (CX-4), reaching *IY* and *RRA* of 100%. At the optimal conditions (contact time 49 min, pH 6.73, and [ASNase] 0.26 mg·mL^−1^), the ASNase-CXs biocomposite was characterized and evaluated in terms of kinetic properties and operational, thermal, and pH stabilities. The immobilized ASNase onto CX-4 retained 71% of its original activity after six continuous reaction cycles, showed good thermal stability at 37 °C (*RRA* of 91% after 90 min), and was able to adapt to both acidic and alkaline environments. Finally, the results indicated a 3.9-fold increase in the immobilized ASNase affinity for the substrate, confirming the potential of CXs as a support for ASNase and as a cost-effective tool for subsequent use in the therapeutic and food sectors.

## 1. Introduction

L-asparaginase (ASNase, EC 3.5.1.1) is a biocatalyst widely used in industrial applications in a fast-growing global market [1]. ASNase is mainly used in food, therapeutic, and biosensing applications. Its primary mechanism of action is based on the hydrolysis of the amino acid L-asparagine into L-aspartic acid and ammonia. In the food industry, ASNase can reduce the production of acrylamide, a carcinogenic substance, without changing the flavor [2]. Acrylamide formation occurs mainly in starch-rich foods due to the Maillard reaction [3]. However, as food composition can affect enzyme activity [2], the ideal ASNase to be used in the food industry must fulfill some requirements, such as improved stability (over a wide range of temperatures and pH), high substrate specificity, and conversion rate [4]. These properties are essential for reducing processing time and costs [5]. Several ASNases were already used to reduce the acrylamide levels in some food products, such as potatoes [6], bread [7], French fries [3], coffee [8], and biscuits [9], showing promising reductions of 99% in acrylamide levels in some cases. In these cases, ASNase is normally dissolved in water and added directly to the food products as a pre-treatment. Ultimately, the enzyme is deactivated during the heating process, ensuring its safe application [2]. ASNase has received “generally recognized as safe” status from the US government and a favorable evaluation as a food additive by the Joint FAO/WHO Expert committee [10]. However, a cost-effective, highly efficient, and stable solution remains to be found [11].

Another important application of ASNase is as an antitumor biopharmaceutical. Acute lymphoblastic leukemia (ALL) is the most common type among children. Considerable progress has been made in the therapeutic sector regarding leukemia treatment with the development of recombinant enzymes, namely ASNase, which has shown promising antineoplastic properties. Unlike normal cells, leukemic cells have little or no asparagine synthetase and, consequently, cannot produce L-asparagine, an essential amino acid for leukemic cells [12]. Therefore, the presence of ASNase deprives tumor cells from this important growth factor, leading to cell death [13]. Currently, ASNases available for pharmaceutical applications are produced from bacteria. However, this type of ASNase may cause adverse side effects, such as fever, anaphylaxis, and hypersensitivity reactions [14]. In addition, the purification processes required are costly, limiting their availability for industrial processes [15]. Although PEGylation reduces the immunogenicity of ASNase, neutralizing hypersensitivity reactions still occur [16]. ASNase loading onto red blood cells (RBCs) is also a promising strategy for reducing immunological adverse reactions; however, problems such as the partial disruption of the cell membrane may result in irreversible changes in both physical and chemical properties of RBCs [17]. Therefore, the search for ASNases with enhanced stability can drastically improve their catalytic performance and cost effectiveness by allowing their reuse over several cycles. Enzyme immobilization on solid supports is known to contribute to these types of improvements [18,19]. Different types of supports, including inorganic, organic, and hybrid materials, have been reported in the literature for ASNase confinement [1,20]. Carbon-based nanomaterials have been successfully used for enzyme immobilization due to their unique porous structure and size, the possibility of introducing numerous functional groups, high surface area, adsorption capacity, and biocompatibility, offering interesting properties for catalytic applications [21,22]. Carbon gels are novel and promising carbon-based nanomaterials for the immobilization of proteins [23]. These materials are produced by carbonizing organic gels obtained by polymerization reaction between resorcinol and formaldehyde, using water as the solvent and a basic catalyst as the reaction promoter [24,25]. Carbon gels can be divided into aerogels, xerogels, or cryogels depending on the drying method used in their production (supercritical drying, conventional drying, and freeze-drying, respectively). The main feature of a carbon xerogel (CX) is the possibility of tailoring its mesoporosity (pores with an average size between 2 and 50 nm) and macroporosity (pores with an average size higher than 50 nm) during the synthesis process by selecting suitable conditions such as solution pH, type, and amount of solvent, reactants concentration, catalyst type, temperature, and time of synthesis, among others [26]. CX porosity can be customized with relatively high precision by adjusting all the variables involved in the synthesis step. This great advantage allows the design of tailored porous textures for specific applications. The CXs’ use for drug conjugation was already reported with high adsorption capacities [27], as well their biocompatibility [28], showing the CXs’ promising ability to be used as ASNase carriers.

Despite CX’s promising properties, no works about ASNase or other enzyme immobilization onto these families of materials are found in the literature. This work’s primary goal is the development of a novel ASNase-CX bioconjugate through optimization of ASNase immobilization conditions by physical adsorption onto CXs. The affinity and interactions of a commercial ASNase from *Escherichia coli* with CXs with different pore sizes were studied. The immobilization conditions were optimized by evaluating different pH, immobilization times, and enzyme concentrations for maximizing the immobilization yield (*IY*) and the enzyme relative recovered activity (*RRA*). For this purpose, an in-depth central composite experimental design and response surface methodology were used to account for not only the effects of each of the studied factors on the response but also the influence of their interactions, while reducing the number of necessary experiments. The effectiveness of the immobilization was confirmed by the characterization of the ASNase-CX bioconjugate. Finally, the operational, thermal, and pH stabilities, as well as the kinetic parameters of the immobilized ASNase, were determined.

## 2. Materials and Methods

### 2.1. Free and Immobilized ASNase Properties

Lyophilized (with no additives) and purified type II ASNase from *E. coli* (P1321-10000; 10,000 IU) were supplied by Deltaclon S.L., Spain. Carbon xerogels with different pore sizes, namely 4, 13, and 30 nm (CX-4, CX-13, and CX-30, respectively), were prepared by polycondensation of resorcinol and formaldehyde, using different pH values (6.0, 5.2, and 5.0, respectively), according to a procedure already described in detail [27]. L-asparagine (≥99.0%), tris(hydroxymethyl)aminomethane (TRIS) (≥99.0%) and disodium hydrogen phosphate (≥99.0%) were purchased from VWR International, LLC. Trichloroacetic acid (TCA) (≥99.0%) was obtained from J.T. Baker. Citric acid (≥99.5%) and Nessler’s reagent (potassium tetraiodomercurate (II)) were supplied by Merck Chemical Company (Darmstadt, Germany). Nessler’s reagent is an acutely toxic substance that can be fatal by ingestion, skin absorption, or inhalation. Only trained personnel with appropriate training and proper personal protective equipment (PPE) should handle this material. Sodium hydroxide (≥98.0%) and hydrochloric acid (37%) were acquired from Sigma-Aldrich.

### 2.2. Characterization Techniques

The CX textural properties were evaluated by N_2_ adsorption-desorption at −196 °C on a Quantachrome NOVA 4200e instrument. Before analysis, each sample (~100 mg) was degassed at 160 °C, 3 h, under vacuum. The specific surface area (*S_BET_*) was calculated by multipoint Brunauer-Emmett-Teller (BET) analysis of the obtained isotherm in the relative pressure range 0.05–0.15 [29], the total pore volume (*V_P_*) was determined at *P*/*P*_0_ = 0.98, and the average mesopore width (*L*) was determined by the Barrett-Joyner-Halenda (BJH) method [30].

The point of zero charge (*pH_PZC_*) was determined according to a method proposed by Rivera-Utrilla et al. [31]. In brief, Erlenmeyer flasks containing 20 mL of 0.01 M NaCl were prepared, and their pH was adjusted with the addition of 0.1 M NaOH or HCl to the desired values (between 2 and 11). Once the pH stabilized, 20 mg of CXs was added, and the Erlenmeyer flasks were shaken for 20 h in a magnetic stirrer (VWR). Blank tests were carried out with no CXs to eliminate other influences. After shaking, the pH of the blank test was measured and defined as *pH_initial_*. The *pH_PZC_* value is where the curve *pH_final_* vs. *pH_initial_* crosses the line *pH_initial_ = pH_final_* [27].

Scanning electron microscopy (SEM) was performed using a high-resolution field-emission SEM (HR-FESEM) Hitachi SU70 microscope operated at an accelerating voltage of 15 kV. Samples for microscopy analysis were prepared by placing a drop of the suspended CX or the CX with immobilized ASNase on a glass slide and then allowing the solvent to evaporate. A thin carbon film was deposited immediately prior to the sample analysis to ensure sample conductivity.

Thermogravimetric (TG) analyses were carried out using an STA 490 PC/4/H Luxx 134 Netzsch thermal analyzer. For each test, approximately 10 mg of CX was loaded on the sample crucible and heated at 10 °C min^−1^ from 50 °C to 900 °C under air flow, while the weight was measured and recorded continuously. The TG profiles resulted from the average of three independent assays, with a maximum deviation of 5%.

Raman spectra were obtained using a WITec alpha300 R—Confocal Raman Imaging Microscope with a laser wavelength of 532 nm and 20 mW laser power.

### 2.3. ASNase Immobilization over CXs

The ASNase immobilization by physical adsorption onto CXs with different pore sizes was studied by adding 2 mg of each CX to 200 μL of ASNase solution (0.02–0.38 mg·mL^−1^) in an appropriate buffer solution (citrate/phosphate buffer, 50 mM). The immobilization was performed by stirring the mixtures during a particular time in a multifunctional tube rotator (Grant Instruments Lda., model PTR-35), followed by sample centrifugation at 136× *g* for 6 min to separate the CXs from the supernatant. A control was also prepared using free ASNase (without CX) at the studied concentrations in each evaluated buffer. The experimental conditions (contact time, pH, and enzyme concentration) were studied using a Central Composite Design of Experiments to maximize the immobilized *ASNase activity* and the immobilization yield.

### 2.4. ASNase Activity Measurement

The enzyme activity was determined by quantifying ammonia released after L-asparagine (substrate) hydrolysis by ASNase. The experimental procedure comprises the mixture of 50 µL of L-asparagine (189 mM) with 50 µL of initial free enzyme solution (0.02–0.38 mg·mL^−1^), or 50 µL of the supernatant after immobilization, or with 2.0 mg of immobilized ASNase on CXs, in 500 µL of TRIS-HCl buffer (50 mM and pH 8.6), and 450 µL of deionized water, at 37 °C for 30 min under stirring, according to the procedure used in previous studies of the research group [22]. After incubation, the reaction was stopped for the free enzyme and immobilized enzyme by adding 250 µL of TCA 1.5 M. Subsequently, 100 µL of the previous free enzyme solution or 100 µL of the supernatant were mixed with 2.15 mL of deionized water and 250 µL of Nessler’s reagent to measure the amount of produced ammonia [32]. After 30 min of incubation at room temperature, the increase in absorbance was measured by UV-spectroscopy at 436 nm, using a JASCO V-560 UV-Vis spectrophotometer. A calibration curve was previously established using ammonium sulfate. Note that, due to restrictions in the use of mercury-containing agents by some institutions, instead of the proposed method, alternative colorimetric, HPLC [32] or LC-MS/MS-based [33] methods could also be used to perform the *ASNase activity* measurements.

One unit of free *ASNase activity* corresponds to the amount of enzyme that releases 1 µmol of ammonia per minute per volume of enzyme at 37 °C (Equation (1)) [22]:

(1) $$ASNase\;activity\;\left(\frac{U}{\mathrm{mL}}\right)=\frac{\left[NH_{3}\right]\left(\frac{\mathrm{µmol}}{\mathrm{mL}}\right)\times V_{Nessler}\left(\mathrm{mL}\right)\times f_{d}}{t_{r}\left(\min\right)\times V_{Enzyme}\left(\mathrm{mL}\right)}$$

where *V_Nessler_* is the volume of the Nessler solution, *f_d_* is the sample dilution factor, *t_r_* is the reaction time, and *V_Enzyme_* is the volume of the enzyme solution.

One unit of immobilized *ASNase activity* is described as the amount of enzyme that releases 1 µmol of ammonia per minute and per mass of support at 37 °C (Equation (2)) [22].

(2) $$ASNase\;activity\;\left(\frac{U}{\mathrm{mg}}\right)=\frac{\left[NH_{3}\right]\left(\frac{\mathrm{µmol}}{\mathrm{mL}}\right)\times V_{Nessler}\left(\mathrm{mL}\right)\times f_{d}}{t_{r}\left(\min\right)\times m_{s}\left(\mathrm{mg}\right)}$$

where *m_s_* is the mass of the support.

The immobilization yield, *IY* (%), is defined as the difference between the free enzyme activity before immobilization and the activity of the free enzyme remaining in the supernatant after immobilization divided by the free enzyme activity before immobilization (Equation (3)) [22].

(3) $$IY\;\left(\%\right)=\frac{Free\;ASNase\;Activity\left(\frac{U}{\mathrm{mL}}\right)-Supernatant\;ASNase\;Activity\;\left(\frac{U}{\mathrm{mL}}\right)}{Free\;ASNase\;Activity\;\left(\frac{U}{\mathrm{mL}}\right)}\times 100\;$$

The relative recovered activity, *RRA* (%), of the immobilized enzyme was calculated as the ratio between the activity of the effectively immobilized enzyme and the maximum theoretical activity that would exist if the free enzyme was totally immobilized (Equation (4)) [22].

(4) $$RRA\;\left(\%\right)=\frac{Immobilized\;ASNase\;activity\left(\frac{U}{\mathrm{mg}}\right)}{Maximum\;ASNase\;activity\left(\frac{U}{\mathrm{mg}}\right)\;}\times 100$$

where: $Maximum\;ASNase\;activity\;\left(\frac{U}{\mathrm{mg}}\right)=\frac{{\left[NH_{3}\right]}_{free\;ASNase}\left(\frac{µmol}{\mathrm{mL}}\right)\times V_{Nessler}\left(\mathrm{mL}\right)\times f_{d}}{t_{r}\left(\min\right)\times m_{s}\left(\mathrm{mg}\right)}$.

### 2.5. Central Composite Design of Experiments for the Optimization of ASNase Immobilization Conditions

Central composite design of experiments is a factorial design with center points increased by a group of axial points (star points) perfect for estimating a curvature. The distance from a factorial point to the center of the design space is ± 1 for each factor, and the distance from a star point to the center of the design space is ± α, where $\left|\alpha \right|$ > 1. The number of factors used dictated the exact value of α [34]. Thus, the central composite design includes 5 levels per factor, often being used for the response surface methodology (RSM).

In this work, a central composite design of experiments with 19 experiments (three factors, five levels, and five repetitions in the center of the domain) was used to study the influence of contact time, pH, and enzyme concentration on the *IY* and *RRA* of the immobilized ASNase over CXs. The factors and levels were selected based on previous studies using carbon nanotubes as an ASNase immobilization support [22]. The low level (−1), high level (+1), the middle point (0), and star points (−1.68 and +1.68) of this experimental design are defined in Table S1 in the Supporting Information. A detailed experimental plan is given in Table S2 in the Supporting Information. This experimental design methodology allows the simultaneous analysis of the influence of different parameters, extending the evaluation to the combined effect of the factors on the desired response.

The experimental central composite design, analysis of variance (ANOVA), and 3D RSM were carried out using Statistica v.7.0 (Statsoft Inc., Tulsa, OK, USA). Equation (5) describes the regression model of the present system, which includes the following interaction terms:

(5) $$Y=\beta_{0}+\beta_{1}X_{1}+\beta_{2}X_{2}+\beta_{3}X_{3}+\beta_{12}X_{1}X_{2}+\beta_{13}X_{1}X_{3}+\beta_{23}X_{2}X_{3}+\beta_{11}X_{1}^{2}+\beta_{22}X_{2}^{2}+\beta_{33}X_{3}^{2}$$

where *Y* is the predicted response, in this case, the immobilized ASNase relative recovered activity, and *X*_1_, *X_2_,* and *X*_3_ are the coded levels of the independent factors, namely time, pH, and enzyme concentration. The regression coefficients are *β*_0_ (the intercept term); *β*_1_, *β*_2_ and *β*_3_ (the linear coefficients); *β*_12_, *β*_13_, and *β*_23_ (the interaction coefficients); and *β*_11_, *β*_22_, and *β*_33_ (the quadratic coefficients). This model evaluates the effect of each independent factor on the response.

### 2.6. Operational Stability of Immobilized ASNase

The operational stability of ASNase-CXs bioconjugate (0.26 mg·mL^−1^ of ASNase, 49 min of contact time between the enzyme and CX-4, pH 6.7) was assessed by incubating 2 mg of the immobilized ASNase with 50 µL of L-asparagine (189 mM) in 500 µL of TRIS-HCl buffer (50 mM and pH 8.6), and 450 µL of deionized water, at 37 °C for 30 min under stirring. At the end of each cycle, the reaction was stopped by the supernatant removal and the subsequent addition of 250 µL of TCA 1.5 M. The immobilized ASNase was washed twice with phosphate buffer pH 7.0 (±500 µL each wash) and resuspended in a fresh substrate solution to begin the next cycle. Six reaction cycles were carried out, and triplicate runs were made for each assay, and the standard deviation was determined.

### 2.7. Thermal Stability of Free and Immobilized ASNase

The thermal stability of free and immobilized ASNase was investigated by incubating the free and immobilized enzyme in phosphate-citrate buffer pH 7.0 (50 mM) at different temperatures (25 °C, 37 °C, 50 °C, 55 °C, and 60 °C). For this purpose, 2 mg of immobilized enzyme or 50 µL of free ASNase (0.26 mg·mL^−1^) were incubated in a water bath with temperature control for 60 min. The initial enzyme activities were compared with the final activities. For each assay, duplicate runs were made, and the standard deviation was determined.

### 2.8. pH Stability of Free and Immobilized ASNase

To evaluate the pH stability, the free and immobilized ASNase were incubated at different buffers (phosphate-citrate buffer 50 mM for pH values from 4.0 to 7.0, phosphate buffer 50 mM for pH 8.0, and carbonate buffer 50 mM for pH 9.0). After every 30 min, a sample was collected, and the residual activities of free and immobilized ASNase were measured for a total incubation time of 120 min. The relative activity was calculated from every sample’s residual activity to initial activity ratio. For each assay, duplicate runs were made, and the standard deviation was determined.

### 2.9. Enzymatic Kinetic Parameters

The kinetic behavior of free and immobilized ASNase was analyzed according to the Hill equation (Equation (6)) [35]:

(6) $$v=\frac{v_{max}{\left[S\right]}^{n}}{S_{50}^{n}+{\left[S\right]}^{n}}\;$$

where *S*_50_ is the substrate concentration (*S*) at which the initial reaction rate (*v*) is equal to 50% of the maximum reaction rate (*v_max_*) and *n* is the Hill coefficient.

The kinetic parameters *S*_50__,_ *v_max_* and *n* were determined by measuring the *ASNase activity* using L-asparagine as the substrate over a 0.5–750 mM range of initial concentrations. The parameter values were obtained by nonlinear curve fitting of the plot of reaction rate versus substrate concentration using CurveExpert software. The ASNase efficiency was determined by the ratio of *k_cat_* (turnover number) to *S*_50_. *k_cat_* was calculated by dividing *v_max_* by the ASNase total concentration [36].

## 3. Results and Discussion

### 3.1. CX Characterization

The physical–chemical and textural characterizations of CX samples are given in Table 1. The results showed that the different pH of sol-gel processing (6.0, 5.2, and 5.0 for CX-4, CX-13, and CX-30, respectively) resulted in carbon materials with different textural properties. On the one hand, the specific surface area increased with pH. On the other hand, the size of the mesopores decreased. These results are in line with previous reports [37,38] where was also observed that a lower concentration of base catalyst, in other words, lower pH values during sol-gel processing, resulted in carbon materials with larger pore diameters. Overall, the BET surface area decreased in the order CX-30 < CX-13 < CX-4, from 670 to 594 m^2^·g^−1^. The total pore volume (*V_p_*) was very similar for the CX-4 and CX-13 materials (0.91 and 0.92 cm^3^·g^−1^, respectively), increasing to 1.42 cm^3^·g^−1^ for CX-30. The points of zero charge (*pH_PZC_*) obtained for CX-4 and CX-13 were c.a. pH 6. The CX-30 surface was revealed to be slightly more acidic, with a *pH_PZC_* of 5.4, probably due to carboxylic acid surface groups generated during the sol-gel process under more acidic conditions [39].

**Figure 1:**
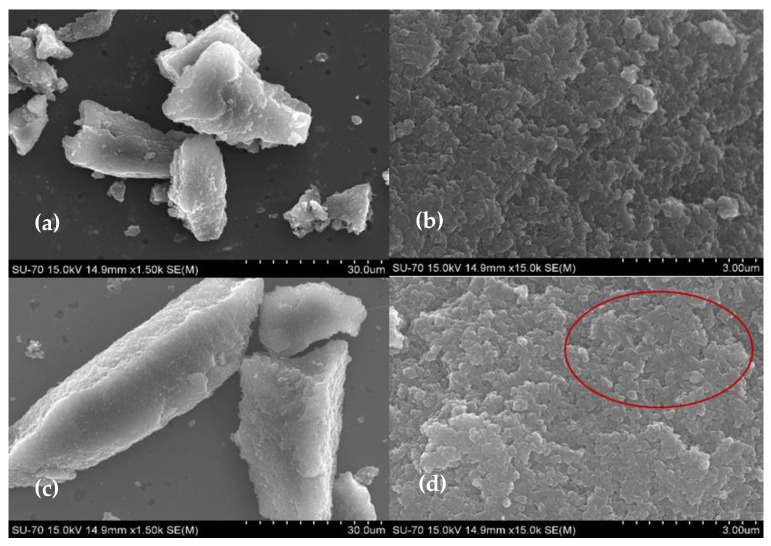
SEM images of CX-4 (**a**,**b**) and ASNase-CX-4 bioconjugate (**c**,**d**).

To confirm the effective immobilization of the enzyme on the material, the CX and the bioconjugate morphology were evaluated by SEM using the optimized ASNase immobilization conditions (0.26 mg.mL^−1^ of ASNase, 2 mg of CX-4, pH 6.7, 49 min of contact time). Figure 1 shows that CX-4 particles are large non-homogeneous polymeric structures with irregular morphology (Figure 1a), consisting of a cluster of several interconnected spherical nanosized particles (Figure 1b), typical of CX structures [40]. After ASNase adsorption, the morphology of the CX particles remains intact (Figure 1c), very similar to their original structures (Figure 1a), meaning that, possibly, the ASNase adsorbs mainly in the pores of the material. These results show that the entire enzyme immobilization procedure did not damage the CX structure, highlighting that the materials would be ready for further enzyme desorption and reuse. Nevertheless, through Figure 1d, an additional organic layer between the spherical particles seems to be observed (red circle), attributed to adsorbed ASNase.

**Figure 2:**
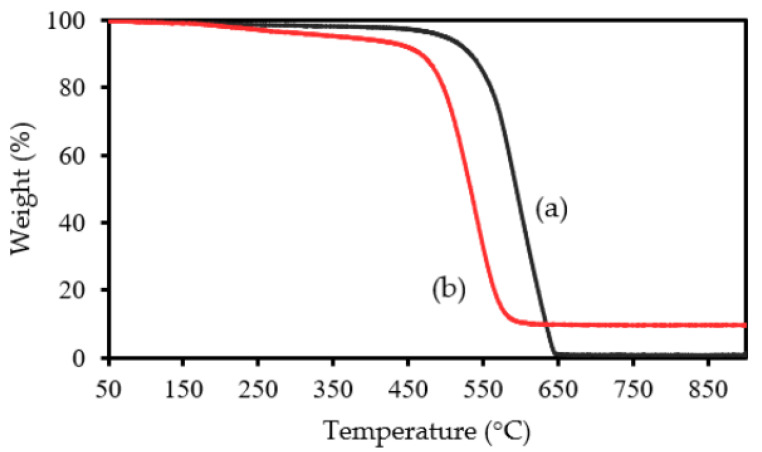
TG analysis of CX-4 (a) before and (b) after ASNase immobilization.

Through TG analysis, we tried to confirm ASNase immobilization onto CX-4. In the thermogram of the original CX-4 (without enzyme, Figure 2a), there was practically no weight loss up to c.a. 500 °C, the carbon material being practically completely gasified above 650 °C. After this temperature, a plateau is reached, corresponding to 1% of the initial mass. This value represents the presence of ashes resulting from the pyrolysis of impurities. The TG profile of the ASNase-CX bioconjugate (Figure 2b) reveals its first weight loss at about 200 °C, which corresponds to the thermal decomposition of the enzyme [41]. The second weight loss begins at 450 °C and is related to the simultaneous pyrolysis of ASNase and CX, reaching a plateau at a temperature close to 550 °C, corresponding to 8.7% of the initial mass of the enzyme-support complex. This value indicates an enzyme content in the bioconjugate of approximately 7.7 wt% (the difference between the mass loss obtained for the CX and that obtained for the bioconjugate). Note that this was only an estimated value, and only one attempt was performed. However, the critical information to retain is that there is indeed enzyme adsorbed onto the CX, validating the immobilization procedure adopted in this study.

**Figure 3:**
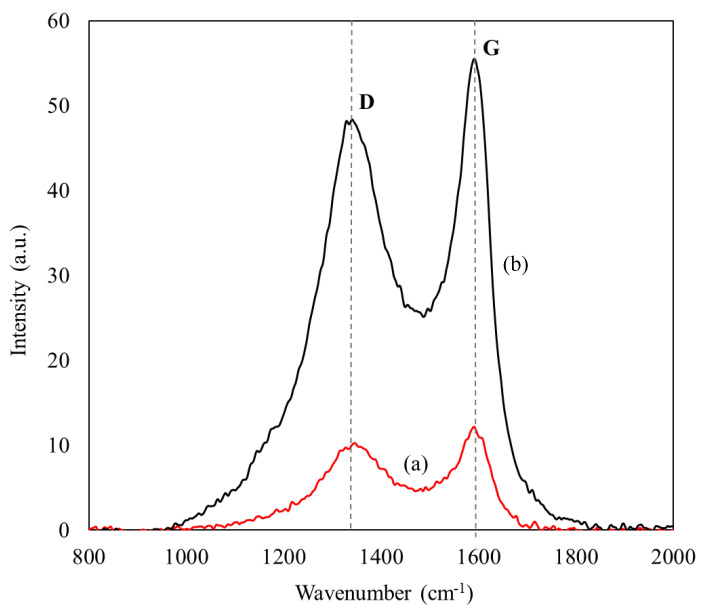
Raman spectra of (a) CX-4 and (b) ASNase-CX-4 bioconjugate.

The degree of disorder in the carbonaceous matrix following enzyme adsorption was investigated using Raman spectroscopy. Figure 3 displays the Raman spectra of CXs and ASNase-CX bioconjugate, which present two broad and overlapping bands in the spectral region between 1000 and 2000 cm^−1^, characteristic of nanographitized carbons. The disorder band (D) emerges at about 1340 cm^−1^ and is related to point defects and disorders in the lattice of the carbon sample, such as openings or doping atoms, as well as crystallite boundaries [42]. The G band, which appears at 1600 cm^−1^, corresponds to the stretch vibration of sp^2^-bonded carbon atoms, which is often associated with a graphitic-like structure [43]. The occurrence of these two bands in CXs has already been reported by other authors [44] and is often ascribed to the increased polarizability of the conjugated π bonds at the sp^2^ sites [45]. The D and G band intensities ratio (I_D_/I_G_) is widely employed to assess disorders in carbonaceous compounds [42,46]. As shown in Table 2, the ASNase adsorption onto CXs promoted a slight increase in the I_D_/I_G_ ratio values, from 1.19 for pristine CXs to 1.35 for the ASNase-CXs composite. This result is indicative of an increase in the structural disorder of CXs after enzyme immobilization, as was already observed during the immobilization of other enzymes onto carbon nanomaterials [47,48].

### 3.2. Optimization of Immobilization Conditions by Experimental Design

The efficiency of the ASNase immobilization onto each CX under study by physical adsorption was optimized by varying the contact time, the solution pH, and the ASNase concentration. The contact time between the enzyme and the support influences the immobilization process, since it affects the probability of interactions between the enzyme molecules and the support surface adsorption sites [49]. The pH influences the superficial charges of both the enzyme and the support and, consequently, the interaction and affinity between them [19]. In turn, the variation of the enzyme concentration allows us to evaluate the maximum amount of enzyme that the support can adsorb. The optimization of these immobilization conditions was carried out using a central composite experimental design by including three factors (time, pH, and enzyme concentration) and five levels, allowing the simultaneous study of the combined effect of the factors on the responses (*IY* and *RRA*). After *IY* and *RRA* determination for each of the 19 experiments, it was possible to notice that the *IY* values were very similar, despite the changes in the experimental conditions. Therefore, experimental analysis was used only to evaluate the influence of the factors on the *RRA* of the immobilized ASNase. The resulting levels of each factor with the *RRA* experimental results for the 19 experiments after ASNase immobilization onto each CX are shown in Table S3 in the Supporting Information.

The central composite experimental design allows for establishing the quadratic model that defines the immobilization system, determining its precision by comparing the lack of fit of model predictions to experimental points with experimental error estimation from the replicates at the central point. The coefficient of correlation (*R^2^*) evaluated the model’s accuracy. Using the experimental data, the second-order polynomial model (Equation (5)) was fitted to the ASNase immobilization results over CX-4, CX-13, and CX-30, obtaining, respectively, Equations (S1)–(S3) in the Supporting Information, in terms of coded factors *X*_1_, *X*_2_, and *X*_3_ (time, pH, and enzyme concentration, correspondingly). The *RRA* values predicted by the models at each experimental point are presented in Table S3 in the Supporting Information.

The statistical significance of the polynomial model for the experimental responses and the considered factors was assessed by analysis of variance (ANOVA) and Pareto charts. The ANOVA results (Table S4 in the Supporting Information) confirm the adequacy of the models to describe the ASNase immobilization onto CX, presenting medium-high correlation coefficients (*R^2^*): 0.7054, 0.93618, and 0.68579 for CX-4, CX-13, and CX-30, respectively. In fact, Table S3 shows that the experimental results are very close to the predicted ones, namely when using CX-13 as the immobilization support. The significance of each model coefficient was evaluated through a *p*-value test, considering 95% confidence, in which low *p*-values (*p* < 0.05) designate a high significance of the corresponding coefficient (values with *p* < 0.05 are red highlighted in Table S4).

Pareto analysis quickly identifies the most statistically important factors affecting the studied response. The factors are arranged in decreasing order, and the effects to the right of the dividing line (factors with *p*-values < 0.05) are significant. Analysis of ANOVA and Pareto charts (Figure S1 in Supporting Information) showed that during the ASNase immobilization onto CX, the most significant factor was the enzyme concentration (*X*_3_) for all the CX under study. In particular, when using CX-4 and CX-13 as immobilization support, the linear and quadratic terms of enzyme concentration were significant, while when using CX-30, only the linear term of enzyme concentration (*X*_3_) was significant. The ASNase immobilization onto CX-13 proved to be the material affected by a higher number of factors, with a marked influence of the linear and quadratic terms of enzyme concentration (*X*_3_), followed by a lower influence of the linear terms of time (*X*_1_) and by the interaction between time and enzyme concentration (*X_1_X*_3_). The linear term of pH (*X*_2_) and the interaction between time and pH (*X_1_X*_2_) also slightly influenced the response. The higher significance of the enzyme concentration factor (*X*_3_) on the *RRA* values verified for all tested materials suggests that this parameter is relevant for the efficiency of the ASNase immobilization over CXs. This factor represents the amount of ASNase molecules available to adsorb onto the CX surface. By varying this parameter, it is possible to reach the highest *RRA* values and, consequently, find the enzyme concentration values that define the saturation point of the material, i.e., the point from which the support has no more active sites available, forming an enzyme layer that covers the entire CX surface [50]. Regarding the influence of the pH factor (*X*_2_) for possible electrostatic interactions, it is known that the isoelectric point (*pI*) of ASNase is between 5.0 and 5.7 [51] and that the points of zero charge of the CXs used in this work vary between 5.4 and 6.1 (Table 1). Therefore, there is a very limited range of pH values where the enzyme and the support have opposite charges, suggesting that electrostatic interactions are not expected to be the most critical interactions responsible for enzyme immobilization and, consequently, the variation of this parameter does not have a notorious influence on the immobilization process. Higher immobilization times were expected to lead to higher *RRA* values. However, the contact time (*X*_1_) between the ASNase and the CXs proved to have almost no influence on the ASNase immobilization process (within the range of tested values). This lack of dependency means that ASNase adsorption onto CXs is very fast, and longer contact times do not significantly improve the results.

The effects of the independent factors (time, pH, and enzyme concentration) and their interaction on the response are represented using RSM. In addition, the optimum values of each factor can be determined, and the response can be predicted. The response surface plots show the *RRA* values reached during the ASNase immobilization onto CX-4, CX-13, and CX-30 as a function of the two factors, while the third factor was kept at a constant level.

The surface plots as a function of pH and time, keeping the ASNase concentration at 0.2 mg·mL^−1^, are represented in Figure S2 in the Supporting Information for the immobilization onto CX-4 (Figure S2a), CX-13 (Figure S2b), and CX-30 (Figure S2c). Figure S2a clearly shows that the critical values of pH and time are within the range of values studied and that, despite the low influence of the pH, it influences the achieved *RRA* values more than the contact time between the enzyme and the support (the higher the surface curvature, the higher the significance of the parameter). On the other hand, Figure S2b shows an increase in *RRA* for higher pH values and lower contact times, possibly not including the optimal conditions for obtaining the maximum *RRA*. Figure S2c suggests that a decrease in contact time results in higher *RRA* values of ASNase when immobilized onto CX-30, with the pH value having only some influence (visible curvature) for higher contact times. In this case, the critical value also seems to be out of the studied range. Figures S3 and S4 complement the analysis of the results, allowing us to verify the optimal conditions for each immobilization support in the study.

The surface plots as a function of enzyme concentration and time for a pH value of 6.0 for the ASNase immobilization onto CX-4, CX-13, and CX-30 are represented in Figure S3a–c, respectively, in the Supporting Information. It was found that the critical value of enzyme concentration to obtain the optimal *RRA* value when ASNase is immobilized onto CX-4 is included in the range of values studied (Figure S3a). The *RRA* values attained were revealed to vary considerably with the ASNase concentration (most significant factor), while the contact time slightly influenced the ASNase immobilization onto CX-4 within the range of values evaluated (low-line curvature). It was also found that the ASNase concentration had a more significant effect on the *RRA* value of the ASNase-CX-13 bioconjugate, as demonstrated by the higher slope of the line at low contact times (Figure S3a,b). Figure S3c does not consider the optimal reaction conditions, meaning that the maximum *RRA* value is likely to be achieved at a pH value other than 6.0. Nevertheless, at pH 6.0, the enzyme concentration affects to a greater extent the *RRA* of the immobilized ASNase onto CX-30 to a higher extent than the contact time.

**Figure 4:**
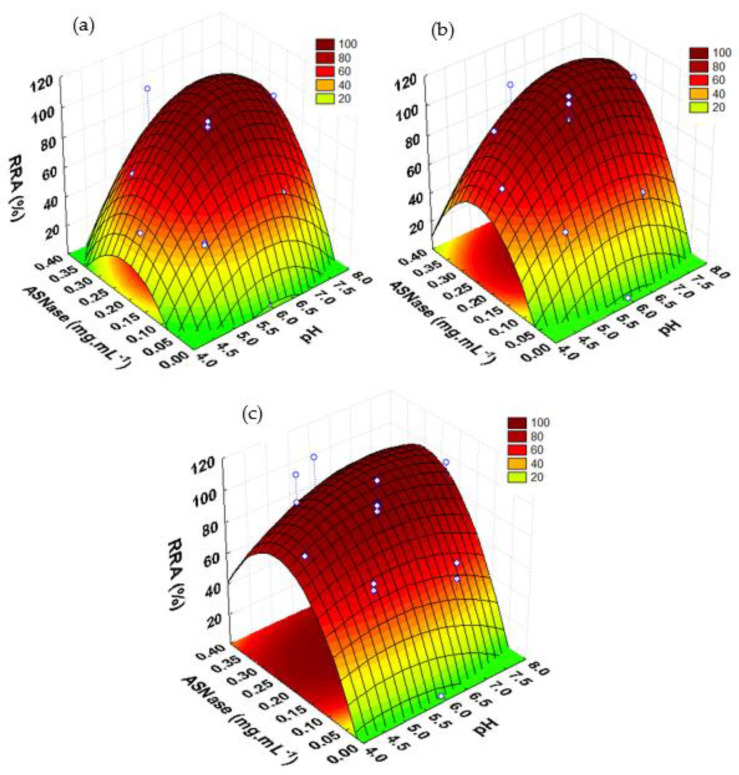
Response surface plots for RRA of immobilized ASNase over CX as a function of enzyme concentration and pH, for a contact time of 60 min. (**a**) CX-4; (**b**) CX-13; (**c**) CX-30.

The surface plots as a function of enzyme concentration and pH for a contact time of 60 min are represented in Figure 4 for the ASNase immobilization onto CX-4, CX-13, and CX-30 (Figure 4a–c, respectively). In all these plots, the critical values of enzyme concentration and pH factors to reach the maximum *RRA* values were well defined. The immobilization process was more efficient at neutral pH values. The lowest *RRA* values were always obtained under acidic conditions (pH 4). Acidic pH values may influence the ASNase stability, causing denaturation. According to Orhan and Uygun [52], both free and immobilized ASNase on magnetic nanoparticles revealed very low activities at pH values of 4.5. The results also showed that the activity of immobilized ASNase was sensitive even to small changes in the enzyme concentration, confirming this to be the most significant factor (with higher curvatures) for the ASNase immobilization process over the three supports.

### 3.3. Critical Values and Model Validation

The Central Composite design methodology, along with the experimental results, allowed optimization of the ASNase immobilization over CXs by predicting each critical parameter value corresponding to the maximum *RRA* (Table S5 in the Supporting Information). Despite the insignificant changes in the *IY* values throughout the experimental results, the efficacy of the immobilization process should be analyzed through a balance between both *RRA* and *IY* values. Therefore, the critical *IY* values were estimated for each of the studied supports, considering the conditions corresponding to the predicted *RRA* critical values (Table S5 in the Supporting Information).

CX with a pore size of 4 nm was the most promising material for ASNase immobilization, driving to total adsorption of ASNase (*IY* of 100%) maintaining its enzymatic activity (*RRA* of 100%), by immobilizing 0.26 mg·mL^−1^ of ASNase in 2 mg of CX-4 for 49 min at pH 6.73 (optimal conditions). This nanomaterial also has the largest specific surface area (Table 1) available to adsorb the enzyme. When using CX-13, total ASNase adsorption (*IY* of 100%) is also predicted, nevertheless with a lower *RRA* value. This could correspond to multi-layer enzyme adsorption not being totally available to react with the substrate. On the other hand, despite the high *RRA* value predicted when using the CX-30 support, the predicted *IY* is very low (*IY* of 1.5%). A possible explanation for this low yield might be related to the lower surface area of CX-30 when compared with the other CXs, suggesting that this material reaches saturation and cannot capture the total amount of enzyme, a large part remaining in the supernatant. The regression model validation was checked by carrying out ASNase immobilization experimental tests under the predicted optimal conditions for each of the supports in the study (Table S5 in the Supporting Information). The excellent agreement verified between the experimental *RRA* and *IY* values and the predicted responses validates the model’s suitability to describe the ASNase immobilization onto CX-4, CX-13, and CX-30.

ASNase immobilization has already been studied over other supports and different immobilization methods [20]. The results of ASNase encapsulation in poly(lactide-co-glycolide) nanoparticles show a preservation of 62.8% of the activity [53]. Similar results were obtained by Zhang et al. [54] while testing natural silk sericin protein microparticles as support for ASNase by covalent attachment. The immobilization of ASNase onto multi-walled carbon nanotubes (MWCNT) by physical adsorption was also analyzed, reporting 90% of *RRA* and *IY* when using pristine MWCNTs [22] and *RRA* of 100% when adsorbed on oxidized MWCNTs, however attaining a lower *IY* (54%) [55]. The results obtained in the present work prove the high potential of using CXs as supports for ASNase immobilization, reaching good values of both *IY* (100%) and *RRA* (100%), namely when using CX with a pore size of 4 nm (CX-4). Considering the advantages of ASNase confinement by a physical adsorption method, the results of this work are considered a promising contribution to the search for a simpler and cost-effective immobilization process for subsequent ASNase application in the therapeutic and food sectors.

### 3.4. Free and Immobilized ASNase Properties

Aiming at real-case application, it is imperative to have a complete insight into the specific properties of the enzyme. Thus, the operational, thermal, and pH stabilities of free and immobilized ASNase were studied under the optimum conditions dictated previously by the factorial experimental design: 0.26 mg·mL^−1^ of ASNase, 49 min of contact time between the enzyme and the CX-4, pH 6.7. Finally, the kinetic parameters were determined.

#### 3.4.1. Operational Stability

**Figure 5:**
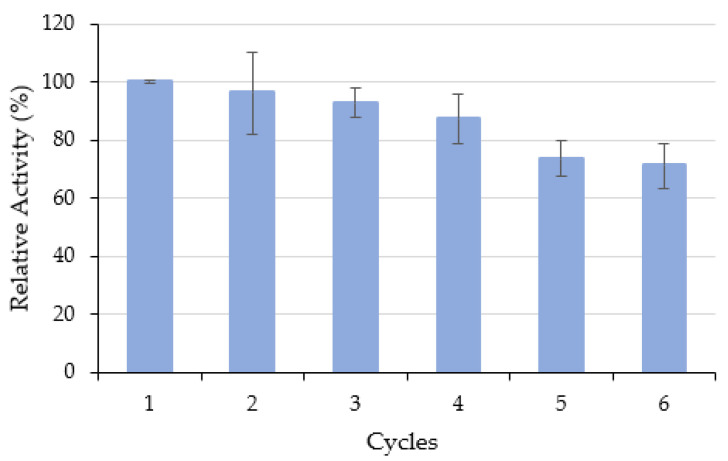
Operational stability of 0.26 mg·mL^−1^ of ASNase immobilized onto 2 mg of CX-4 at pH 6.7 over 49 min of contact time. Error bars represent the standard deviation.

From a commercial point of view, enzyme reusability is a crucial factor, especially when applied on an industrial scale. ASNase immobilization allows enzyme recovery after each reaction cycle and subsequent reuse. The reusability of ASNase-CX bioconjugate was assessed during 6 cycles of L-asparagine hydrolysis and represented as *RRA* vs. cycle (Figure 5), where the *RRA* of the first cycle was set to 100%. The results show that the ASNase-CX bioconjugate may be reused for at least 6 cycles without significant enzyme activity loss. After 6 continuous reaction cycles, the immobilized ASNase retained 71 ± 8% of its initial enzymatic activity. Tarhan et al. [56] reported similar operational stability of ASNase immobilized onto maltose-functionalized magnetic core/shell Fe_3_O_4_@Au nanoparticles, retaining about 78% of ASNase initial activity after 5 reaction cycles. On the other hand, Monajati et al. [57] immobilized ASNase onto functionalized graphene oxide nanosheets by physical adsorption, and at the end of the 6th reuse cycle, the immobilized enzyme showed less than 40% of its original activity. Even trying immobilization by covalent binding, the same authors reported that ASNase could still retain only about 50% of its initial activity. These results highlight the adequacy of the ASNase immobilization by simple physical adsorption onto the pristine CX used in this work, maintaining an enzyme activity comparable to an ASNase immobilized in surface-modified materials by covalent bonding, and without significant enzyme leaching by desorption during continuous use. The slight decrease in activity observed in this work upon repeated usage might be due to the distortion of the active site promoted by the frequent interaction between the substrate and the immobilized enzymes [58].

#### 3.4.2. Thermal Stability

**Figure 6:**
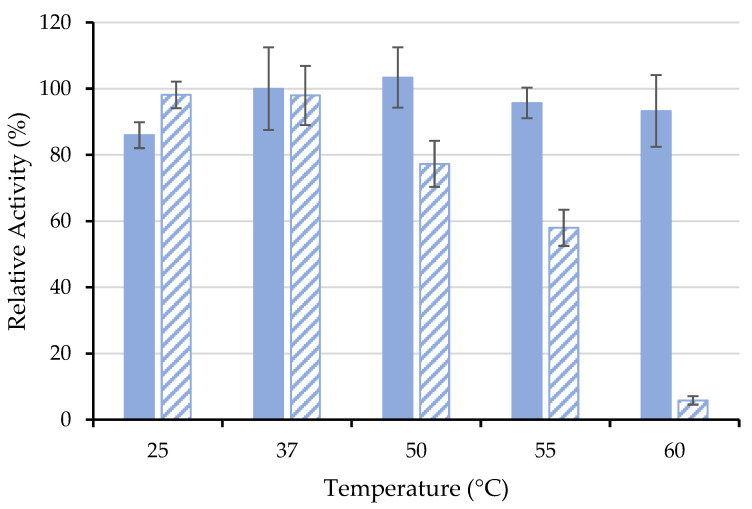
Thermal stability of 0.26 mg·mL^−1^ of (■) free and (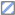) immobilized ASNase onto CX-4 by physical adsorption (pH 6.7, 49 min of contact time) at different temperatures for an incubation time of 60 min. Error bars represent the standard deviation.

Another important characteristic of industrial applications is the thermal stability of enzymes. In this study, the thermal stability of free ASNase and ASNase-CX-4 bioconjugate was investigated for a temperature range between 25 and 60 °C (Figure 6), where the *RRA* at time 0 min was set to 100%.

Usually, the immobilization process is expected to improve the enzyme thermal tolerance due to the protective microenvironment provided by the support, reducing drastic enzyme conformational changes against temperature or even its denaturation [59]. For example, Noma et al. [60] reported that p(HEMA-GMA)-ASNase retained 66% of its initial activity, while free ASNase retained only 18% after incubation at 60 °C for 3 h. Nevertheless, in this work, the free ASNase proved to be more resistant than immobilized one at elevated temperatures (50–60 °C) (Figure 6). The immobilization procedure by physical adsorption involves only physical interaction between the CX and the ASNase, not preventing the denaturation of the enzyme, leading to a decrease in its relative activity. On the other hand, the immobilized ASNase showed similar activity to its free form at temperatures of 25 and 37 °C (human body temperature) without loss of activity in the considered time range (Figure 6). In 2018, Golestaneh and Varshosaz [61] reported ASNase immobilization onto silica nanoparticles through two distinctive cross-linking agents, namely 1-ethyl-3-(3-dimethylaminopropyl)carbodiimide hydrochloride (EDC) and glutaraldehyde, and the results indicate that, at 37 °C, the immobilized ASNase kept 80% and 72% of its enzymatic activity, respectively, after 60 min. Considering the preserved activity at physiological temperatures obtained in the present work, the use of the bioconjugate ASNase-CX may be considered a valuable possibility for the therapeutic applications of ASNase.

#### 3.4.3. Influence of pH

**Figure 7:**
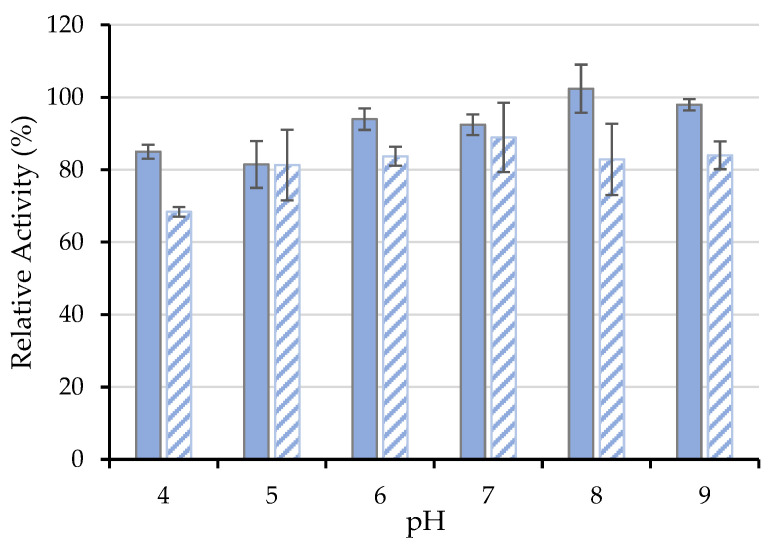
pH effect on the activity of 0.26 mg·mL^−1^ of (■) free and (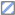) immobilized ASNase onto CX-4 (pH 6.7, 49 min of contact time) for an incubation time of 120 min. Error bars represent the standard deviation.

The effect of several pH conditions, from 4.0–9.0, on free and immobilized *ASNase activity* are shown in Figure 7, where the *RRA* at time 0 min was set to 100%. The free ASNase proved to have no significant activity loss in the range of pH values studied, being more stable at pH 8.0. In turn, the immobilized ASNase revealed similar stability in the pH range between 5.0 and 9.0. Nevertheless, the bioconjugate showed a significant activity decrease at acidic pH values (pH 4.0), suggesting that L-aspartic acid production may act as an ASNase competitive inhibitor under these conditions [62]. Jayaram et al. [63] reported the presence of approximately 4 tight binding sites for L-aspartic acid in ASNase, which is translated into the loss of binding affinity of the enzyme toward the substrate and, hence, a decrease in its catalytic activity at acidic pH. Despite the lower activity values exhibited by the immobilized ASNase at pH 4.0 when compared to the remaining pH values studied, it should be noted that the results shown in Figure 7 are relative activities registered after a given time. The ASNase-CX bioconjugate retained 68% of its initial activity after 120 min of incubation at pH 4.0. The change in the optimal pH noticed between the free and the conjugated ASNase may be related to the immobilization procedure, support structure, or to the enzyme conformational change caused by its binding [64]. According to the obtained results, immobilization onto CX-4 promoted the excellent stability of ASNase in the pH 5.0 to 9.0 range, without significant activity loss concerning the native enzyme. Similar results were obtained by Zhang et al. [54] with no significant changes in the optimal range of pH values between the free and ASNase immobilized onto microparticles of the natural silk sericin protein.

#### 3.4.4. Kinetic Parameters

The kinetics of free and immobilized ASNase onto CX-4 were studied by varying the initial asparagine concentration during the hydrolytic reaction. The enzymatic reaction rate showed a sigmoidal dependency of substrate concentration characteristic of allosteric enzymes (Figure S4 in the Supporting Information). This allosteric sigmoidal behavior is defined by the Hill Equation (Equation (6)), where the *S*_50_ parameter is analogous to the Michaelis-Menten constant (*K_M_*) and the Hill coefficient, *n*, describes the degree of cooperativity between the enzyme subunits and the number of substrate molecules that can bind to the enzyme complex. When *n* is equal to 1, Equation (6) is reduced to the Michaelis–Menten equation. A value of *n* > 1 points to positive kinetic cooperativity (homotropic regulation), meaning that several substrate molecules bind to the enzyme simultaneously. A value of *n* < 1 points to negative kinetic cooperativity, where the binding of a first substrate molecule hampers the attachment of a new one or hinders the catalytic activity of the system [35].

The *S*_50_ values for free and immobilized ASNase were 345 and 89 mM, respectively. Analogously to the Michaelis-Menten constant (*K_M_*), the decrease verified in *S*_50_ for the conjugated ASNase means that the enzyme’s affinity for L-asparagine increased by 3.9-fold as a result of immobilization, also increasing its activity. The L-asparaginase molecule can be extended over the surface of the CX with an improved orientation, leading to a higher availability of the active sites and an enhanced affinity for the L-asparagine [65]. On the other hand, a decrease in the *v_max_* values from 0.050 to 0.012 mM·min^−1^ was verified for free and immobilized ASNase, respectively. The reduction in *v_max_* observed after immobilization is related to the higher mass transfer restriction of the diffusion layer that surrounds the ASNase particles [66], limiting the L-asparagine diffusion toward the ASNase-CX bioconjugate. The enzyme fixation on an immobilization matrix may also reduce the enzyme molecule flexibility, typically reflected in lower catalytic activity [67] and, consequently, in a decrease in the *v_max_* after immobilization. These obtained kinetic parameters led to similar catalytic efficiencies, *k_cat_*/*S*_50_, between free and immobilized ASNase (0.021 and 0.019 mM^−1^min^−1^, respectively). Analogous results were reported by Orhan and Uygun [52] and Ulu et al. [68] for free and immobilized ASNase.

A value over 1 for the Hill coefficient in both free and immobilized ASNase means that both ASNase forms have allosteric regulation with positive kinetic cooperativity. This kinetic behavior exhibited by the ASNase was already reported by Yun et al. [69] with a Hill coefficient of 2.6, which translates into faster catalysis than kinetics without allosteric mechanisms.

## 4. Conclusions

A simple physical adsorption method successfully immobilized a commercial ASNase onto CXs. The Central Composite statistical experimental design was an effective tool to optimize the immobilization conditions, presenting good model accuracies and providing helpful information about the effects of the factors and possible interactions. The parameter that revealed the most significant influence on the immobilization effectiveness was the ASNase concentration, as opposed to the contact time and the pH, which had no considerable impact. Moreover, CX-4 proved to be the most promising support for ASNase immobilization, attaining exceptional *RRA* and *IY* values (100%) under the optimum conditions (contact time of 49 min, ASNase concentration of 0.26 mg·mL^−1^, pH of 6.73). SEM, TG analysis, and Raman spectroscopy confirmed the effective ASNase immobilization onto CX-4. Reusability is one of the main advantages of using immobilized enzymes in biotechnological applications compared with the native form. In this case, the confinement onto CX-4 allowed the ASNase to retain 71% of its original activity after 6 continuous cycles. Apart from the reusability, immobilization allowed ASNase to maintain 91% of its initial activity at 37 °C after 90 min and present good stability in the pH range from 5.0 to 9.0, with relative activities above 80% after 2 hours. Furthermore, the obtained kinetic constants indicate a 3.9-fold increase in the affinity of the immobilized enzyme toward the substrate when compared to the free enzyme. The obtained results show that CXs are promising supports for ASNase, with no chemical modification or covalent binding required, contributing to the search for a more straightforward and cost-effective immobilization process for posterior use in several application fields, e.g., in medicine, pharmaceutical, and food industries.

## Figures and Tables

**Table 1:** Characterization of CX samples: point of zero charge (*pH_PZC_*), surface area (*S_BET_*), total pore volume (*Vp*), and average mesopore width (*L*), obtained by adsorption of N_2_ at −196 °C.

Sample	*pH_PZC_*	*S_BET_* (m^2^·g^−1^)	*V_p_* (cm^3^·g^−1^)	*L* (nm)
CX-4	6.1	670	0.91	3.9
CX-13	6.0	618	0.92	13.7
CX-30	5.4	594	1.42	32.8

**Table 2:** Band D and G intensities of CXs and ASNase-CX bioconjugate calculated from Raman spectra.

Material	Area (a.u. cm^−1^)		I_D_/I_G_
	Band D	Band G	
CX-4	784	657	1.19
ASNase-CX-4	3680	2734	1.35

## Data Availability

The datasets used and/or analyzed during the current study are available from the corresponding author on reasonable request.

## Supplementary Materials

The following supporting information can be downloaded at: https://www.mdpi.com/article/10.3390/biotech11020010/s1, Figure S1: Pareto chart of standardized effects for the Central Composite design for ASNase immobilization onto (a) CX-4, (b) CX-13, and (c) CX-30. (1) time; (2) pH; (3) enzyme concentration. Figure S2: Response surface plots for the *RRA* of immobilized ASNase over CX as a function of pH and time with an enzyme concentration of 0.2 mg·mL^−1^. (a) CX-4; (b) CX-13; (c) CX-30. Figure S3: Response surface plots for *RRA* of immobilized ASNase over CX as a function of enzyme concentration and time at pH 6. (a) CX-4; (b) CX-13; (c) CX-30. Figure S4: Initial reaction rates (*v*_0_) for free (♦) and immobilized ASNase (■) (0.26 mg·mL^−1^) onto CX-4 by physical adsorption. The solid lines represent the experimental data fit to the Hill equation; Table S1: Factor levels for a central composite design to evaluate ASNase immobilization over CX. Table S2: Central composite experimental design plan. Table S3: Central Composite design matrix with the experimental data (Exp.) and predicted (Pred.) values of *RRA* obtained after ASNase immobilization onto CX-4, CX-13, and CX-30, as a function of the coded factors *X*_1_, *X*_2_, *X*_3__,_ respectively time (min), pH, and enzyme concentration (mg·mL^–1^). Table S4: Analysis of variance (ANOVA) for the fitted quadratic polynomial models of *RRA* values obtained after ASNase immobilization onto CX-4, CX-13, and CX-30. Table S5: Experimental and predicted relative recovered activity (*RRA*) and immobilization yield (*IY*) maximum values at critical process conditions for the ASNase immobilization onto carbon xerogels with different pore sizes, namely 4, 13, and 30 nm (CX-4, CX-13, and CX-30, respectively).
