# Supplementary material for: Immobilization and Characterization of L-Asparaginase over Carbon Xerogels
Source: BioTech (Basel). 2022 Apr 14;11(2):10. doi: 10.3390/biotech11020010 (PMC9264400; doi:10.3390/biotech11020010)
Supplement: Supplementary file 1 [file biotech-11-00010-s001.zip › biotech-1660740-supplementary.pdf]

Immobilization and Characterization of L-Asparaginase over Carbon Xerogels

Rita A. M. Barros[1,2,†], Raquel O. Cristóvão[1,2,†] , Sónia A. C. Carabineiro[1,3], Márcia C. Neves[4], Mara G. Freire[4], Joaquim L. Faria[1,2], Valéria C. Santos-Ebinuma[5], Ana P. M. Tavares[4,*] and Cláudia G. Silva[1,2,*]

[1] LSRE-LCM - Laboratory of Separation and Reaction Engineering – Laboratory of Catalysis and Materials, Faculty of Engineering, University of Porto, Rua Dr. Roberto Frias, 4200-465 Porto, Portugal;
[2] ALiCE – Associate Laboratory in Chemical Engineering, Faculty of Engineering, University of Porto, Rua Dr. Roberto Frias, 4200-465 Porto, Portugal;
[3] LAQV-REQUIMTE, Department of Chemistry, NOVA School of Science and Technology, Universidade NOVA de Lisboa, 2829-516 Caparica, Portugal;
[4] CICECO-Aveiro Institute of Materials, Department of Chemistry, University of Aveiro, 3810-193 Aveiro, Portugal;
[5] Department of Engineering Bioprocess and Biotechnology, School of Pharmaceutical Sciences, UNESP-University Estadual Paulista, Araraquara, Brazil;
* Correspondence: C. G. S: cgsilva@fe.up.pt; Tel.: +351 220 414 874; A. P. M. T: aptavares@ua.pt; Tel.: +351 234 401 520
† These authors contributed equally to this work.

Model Equations:

$$Y(CX-4) = -363.27 + 0.17X_1 + 119.92X_2 + 447.25X_3 + 0.11X_1X_2 - 0.83X_1X_3 + 57.71X_2X_3 - 0.01X_1^2 - 10.47X_2^2 - 1501.96X_3^2 \quad \text{(S1)}$$

$$Y(CX-13) = -440.42 + 2.56X_1 + 111.42X_2 + 922.57X_3 - 0.36X_1X_2 - 3.94X_1X_3 + 41.98X_2X_3 - 7.46X_2^2 - 1790.42X_3^2 \quad \text{(S2)}$$

$$Y(CX-30) = 110.83 - 4.80X_1 + 20.78X_2 + 573.48X_3 - 0.24X_1X_2 - 0.17X_1X_3 + 43.50X_2X_3 - 3.60X_2^2 - 1564.96X_3^2 \quad \text{(S3)}$$

Table S1. Factor levels for a central composite design to evaluate the ASNase immobilization over CX.

Factors	Parameters	Coded level				
		-1.68	-1	0	+1	+1.68
X_1	Time (min)	9.6	30.0	60.0	90.0	110.4
X_2	pH	4.32	5.00	6.00	7.00	7.68
X_3	ASNase Concentration (mg·mL^{-1})	0.02	0.09	0.20	0.31	0.38

Table S2. Central composite experimental design plan.

Run	X_1	X_2	X_3
1	-1	-1	-1
2	1	-1	-1
3	-1	1	-1
4	1	1	-1
5	-1	-1	1
6	1	-1	1
7	-1	1	1
8	1	1	1

9	-1.68	0	0
10	1.68	0	0
11	0	-1.68	0
12	0	1.68	0
13	0	0	-1.68
14	0	0	1.68
15	0	0	0
16	0	0	0
17	0	0	0
18	0	0	0
19	0	0	0

Table S3. Central Composite design matrix with the experimental data (Exp.) and predicted (Pred.) values of *RRA* obtained after ASNase immobilization onto CX-4, CX-13 and CX-30, as a function of the coded factors X_1, X_2, X_3, respectively time (min), pH and enzyme concentration (mg·mL^{-1}).

**Run**	X_1 **(min)**	X_2	X_3 **(mg·mL^{-1})**	**Relative Recovered Activity (%)**					
				**CX-4**		**CX-13**		**CX-30**	
				**Exp.**	**Pred.**	**Exp.**	**Pred.**	**Exp.**	**Pred.**
1	30.0	5.00	0.09	43.0	42.1	33.3	29.6	70.0	96.9
2	90.0	5.00	0.09	42.4	29.3	47.9	55.3	74.1	45.9
3	30.0	7.00	0.09	51.2	48.0	49.5	59.3	52.6	74.6
4	90.0	7.00	0.09	52.3	48.9	41.3	41.5	62.4	53.0
5	30.0	5.00	0.31	56.8	66.3	83.7	95.2	110.9	134.3
6	90.0	5.00	0.31	33.3	42.6	66.9	68.9	93.6	85.5
7	30.0	7.00	0.31	78.4	97.6	139.0	143.3	89.0	131.2
8	90.0	7.00	0.31	80.6	87.6	58.1	73.5	124.6	111.7
9	9.6	6.00	0.20	95.7	84.0	126.4	119.0	242.2	180.8
10	110.4	6.00	0.20	61.9	64.9	91.1	81.9	80.1	121.7
11	60.0	4.32	0.20	42.0	42.1	68.9	64.3	83.6	82.0
12	60.0	7.68	0.20	93.5	84.8	105.0	93.0	103.5	85.3
13	60.0	6.00	0.02	0.0	15.3	0.0	-2.5	0.0	-0.01
14	60.0	6.00	0.38	91.9	68.1	93.7	79.6	100.5	80.8
15	60.0	6.00	0.20	82.4	93.0	94.6	99.7	110.5	93.8
16	60.0	6.00	0.20	91.8	93.0	93.4	99.7	95.5	93.8
17	60.0	6.00	0.20	65.8	93.0	110.8	99.7	91.3	93.8
18	60.0	6.00	0.20	140.3	93.0	105.8	99.7	94.8	93.8
19	60.0	6.00	0.20	83.2	93.0	90.9	99.7	73.5	93.8

X_1 – contact time (min); X_2 – pH; X_3 – ASNase concentration.

Table S4. Analysis of variance (ANOVA) for the fitted quadratic polynomial models of *RRA* values obtained after ASNase immobilization onto CX-4, CX-13 and CX-30.

Source	Sum of Squares (SS)			df	Mean Square (MS)			*F*-value			*p*-value		
	CX-4	CX-13	CX-30		CX-4	CX-13	CX-30	CX-4	CX-13	CX-30	CX-4	CX-13	CX-30
(1) *X1* (L)	440.36	1660.35	4224.52	1	440.36	1660.35	4224.52	0.7930	10.8903	3.2876	0.3964	**0.0092**	0.1032
X1 (Q)	585.14	0.95	5634.08	1	585.14	0.95	5634.08	1.0538	0.0063	4.3845	0.3314	0.9387	0.0658
(2) *X2* (L)	2208.20	1000.83	12.99	1	2208.20	1000.83	12.99	3.9768	6.5645	0.0101	0.0773	**0.0306**	0.9221
X2 (Q)	1490.42	757.37	175.92	1	1490.42	757.37	175.92	2.6841	4.9676	0.1369	0.1358	0.0528	0.7199
(3) *X3* (L)	3378.00	8133.37	7883.20	1	3378.00	8133.37	7883.20	6.0835	53.3469	6.1348	**0.0358**	**0.0000**	**0.0352**
X3 (Q)	4494.57	6386.71	4879.48	1	4494.57	6386.71	4879.48	8.0943	41.8905	3.7973	**0.0192**	**0.0001**	0.0831
1L by 2L	93.67	944.35	428.50	1	93.67	944.35	428.50	0.1687	6.1940	0.3335	0.6909	**0.0345**	0.5778
1L by 3L	59.78	1351.57	2.42	1	59.78	1351.57	2.42	0.1077	8.8649	0.0019	0.7503	**0.0155**	0.9663
2L by 3L	322.41	170.59	183.18	1	322.41	170.59	183.18	0.5806	1.1189	0.1426	0.4656	0.3177	0.7145
Error	4997.48	1372.16	11564.92	9	555.28	152.46	1284.99						
Total SS	16963.41	21500.03	36806.00	18									

CX-4: R^2 = 0.7054; CX-13: R^2 = 0.93618; CX-30: R^2 = 0.68579. *X1:* Time (min); *X2*: pH; *X3*: ASNase Concentration (mg·mL^{-1}). Df, degrees of freedom; L, linear; Q, quadratic

Table S5. Experimental and predicted relative recovered activity (*RRA*) and immobilization yield (*IY*) maximum values at critical process conditions for the ASNase immobilization onto carbon xerogels with different pore sizes, namely 4, 13 and 30 nm (CX-4, CX-13 and CX-30, respectively).

Sample	Critical Value Conditions			Predicted Values		Experimental Values	
	Time (min)	pH	Enzyme Concentration (mg·mL^{-1})	*RRA* (%)	*IY* (%)	*RRA* (%)	*IY* (%)
CX-4	49.0	6.73	0.26	100	100	97 ± 4	99.2 ± 0.4
CX-13	116	5.18	0.19	85	100	89 ± 2	99.9 ± 0.1
CX-30	67.8	6.90	0.28	100	1.5	100 ± 5	0 ± 8

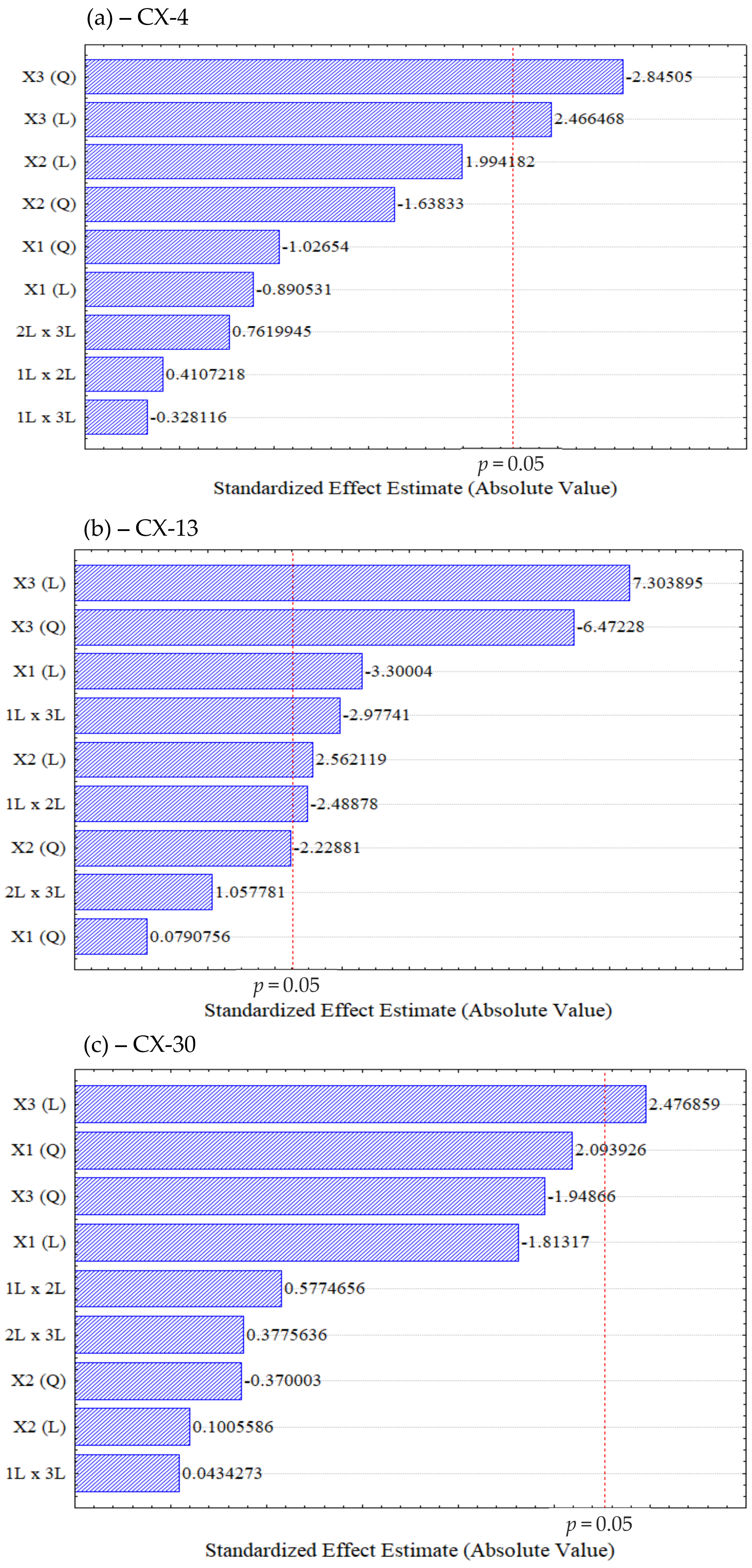

Figure S1. Pareto chart of standardized effects for the Central Composite design for ASNase immobilization onto (a) CX-4, (b) CX-13 and (c) CX-30. (1) time; (2) pH; (3) enzyme concentration.

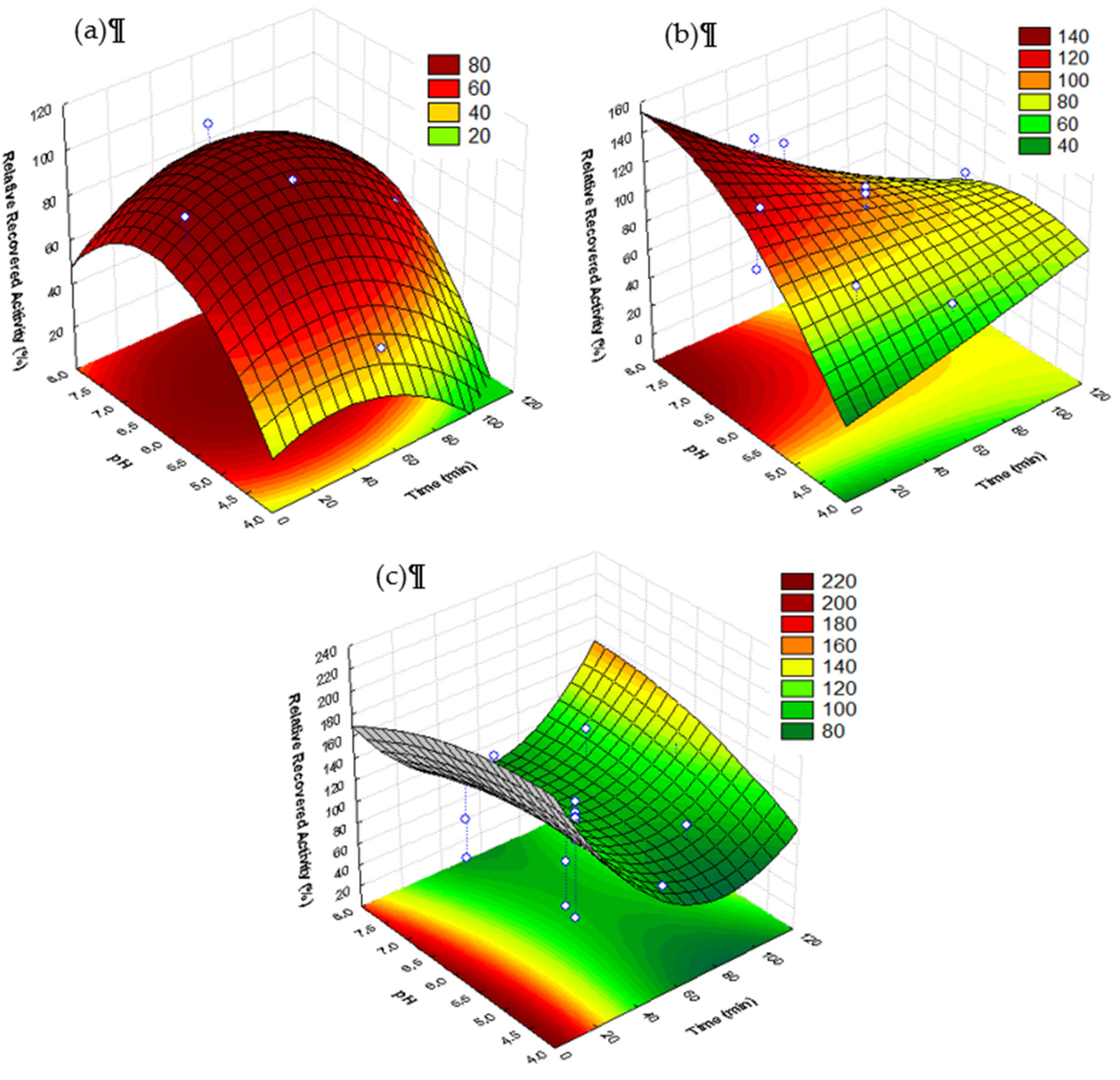

Figure S2. Response surface plots for *RRA* of immobilized ASNase over CX as a function of pH and time with an enzyme concentration of 0.2 mg·mL^{-1}. (a) CX-4; (b) CX-13; (c) CX-30.

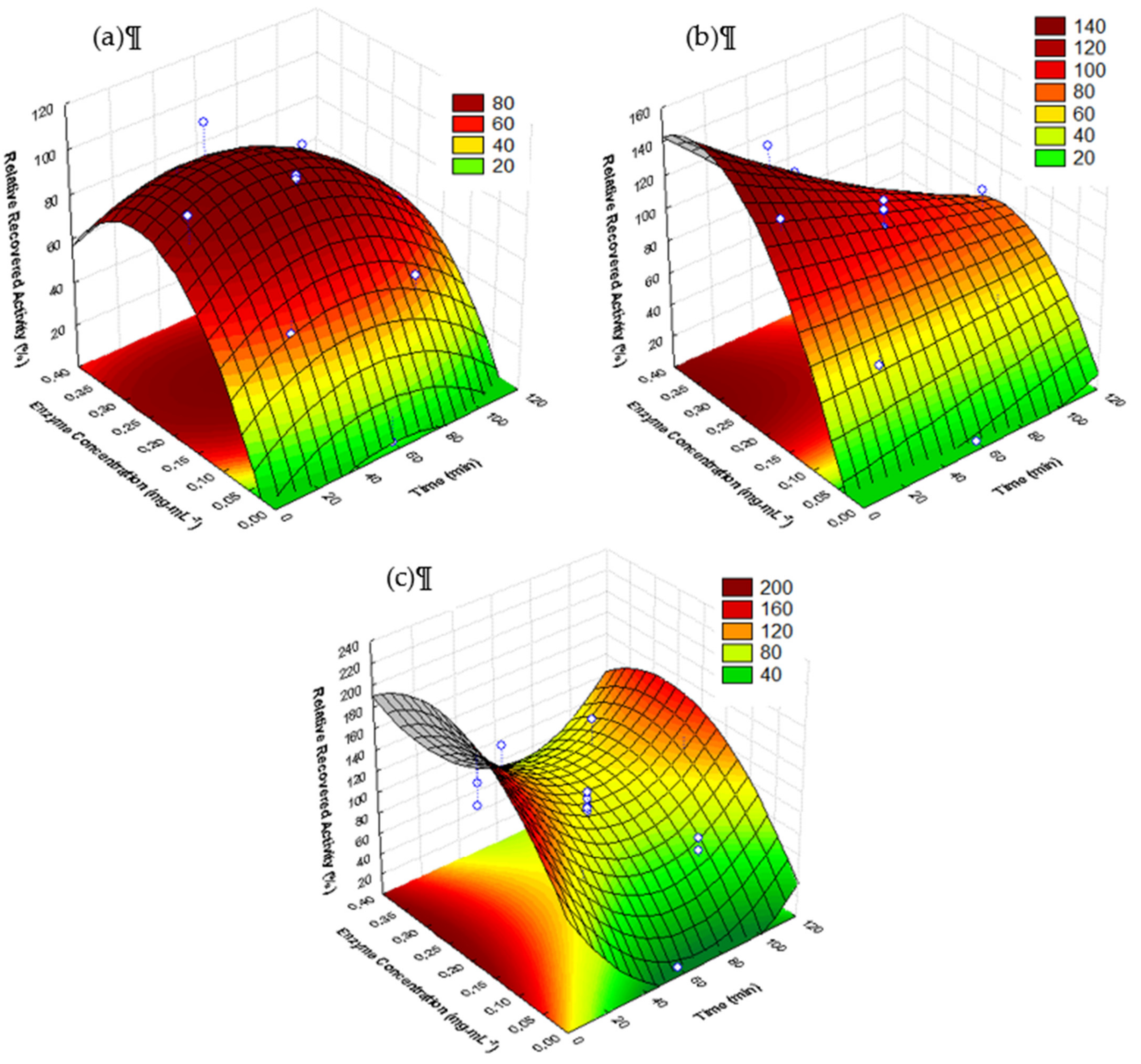

Figure S3. Response surface plots for *RRA* of immobilized ASNase over CX as a function of enzyme concentration and time, at pH 6. (a) CX-4; (b) CX-13; (c) CX-30.

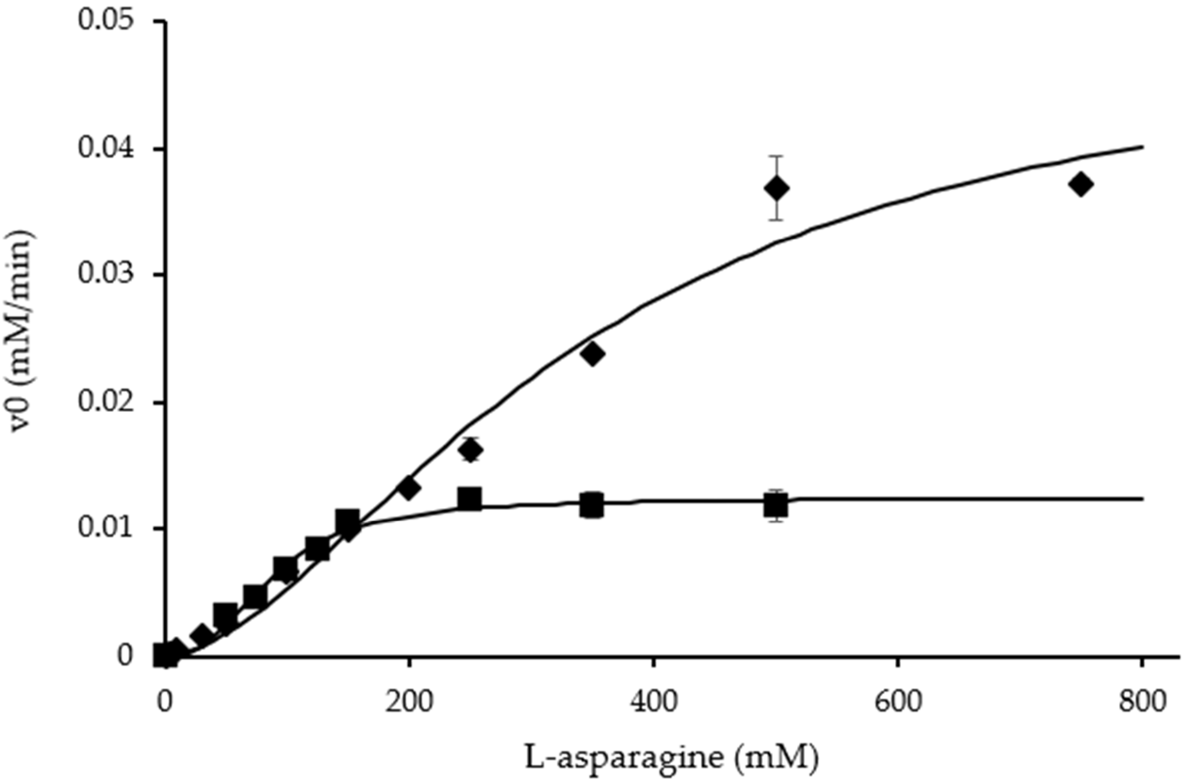

Figure S4. Initial reaction rates (v_0) for free (♦) and immobilized ASNase (■) (0.26 mg·mL^{-1}) onto CX-4 by physical adsorption. The solid lines represent the experimental data fit to the Hill equation.
